# When should meta‐analysis avoid making hidden normality assumptions?

**DOI:** 10.1002/bimj.201800071

**Published:** 2018-07-30

**Authors:** Dan Jackson, Ian R. White

**Affiliations:** ^1^ Statistical Innovation Group AstraZeneca Cambridge UK; ^2^ MRC Clinical Trials Unit at UCL London UK

**Keywords:** central limit theorem, distributional assumptions, normal approximation, random effects models

## Abstract

Meta‐analysis is a widely used statistical technique. The simplicity of the calculations required when performing conventional meta‐analyses belies the parametric nature of the assumptions that justify them. In particular, the normal distribution is extensively, and often implicitly, assumed. Here, we review how the normal distribution is used in meta‐analysis. We discuss when the normal distribution is likely to be adequate and also when it should be avoided. We discuss alternative and more advanced methods that make less use of the normal distribution. We conclude that statistical methods that make fewer normality assumptions should be considered more often in practice. In general, statisticians and applied analysts should understand the assumptions made by their statistical analyses. They should also be able to defend these assumptions. Our hope is that this article will foster a greater appreciation of the extent to which assumptions involving the normal distribution are made in statistical methods for meta‐analysis. We also hope that this article will stimulate further discussion and methodological work.

## 1 INTRODUCTION

Meta‐analysis is commonly used in medical statistics, and other application areas, and now requires little introduction. Simple statistical methods are typically used to perform meta‐analyses, where pooled estimates are calculated as weighted averages. The simplicity of the calculations involved in these methods conceals the distributional assumptions that justify them. Here, we will focus on the normal distribution in order to examine how this distribution is extensively, and often implicitly, used in meta‐analysis. For the majority of the paper, we will focus on the standard methods for meta‐analysis that we anticipate that many readers will already be familiar with. For the less initiated reader, the textbook by Borenstein, Hedges, Higgins, and Rothstein (2009) provides a particularly clear and accessible introduction to this type of methodology.

Let us begin by considering perhaps the simplest possible case. Here, we have a set of independent studies, each of which provides an estimate of a particular treatment effect (or another quantity of interest). We will refer to the estimate from the *i*‐th study as $Y_{i}$, i=1,…,k. The common‐effect model (sometimes referred to as the fixed‐effect model) assumes, to within‐study sampling error, that all studies independently estimate the same true effect μ. Using $\varepsilon_{i}$ to denote this statistical error from the *i*‐th study, we can write the common‐effect model as $Y_{i}=\mu +\varepsilon_{i}$, where $\text{E}(\varepsilon_{i})=0$ and $\text{Var}\left(\varepsilon_{i}\right)=\sigma_{i}^{2}$. The conventional pooled estimate is then $\hat{\mu }=\sum w_{i}Y_{i}/\sum w_{i}$, where $w_{i}$ is the ‘weight’ of the *i*‐th study. These study weights are the reciprocals of the *estimated* variances of the $\varepsilon_{i}$. We will denote these estimated within‐study variances as $s_{i}^{2}$, so that $w_{i}=s_{i}^{-2}$. Standard formulae are available for calculating the $s_{i}^{2}$, and so the weights $w_{i}$, for a wide range of effects and outcomes used in meta‐analysis (Borenstein et al., 2009). At this point, we ask the reader to stop and reflect on a question before reading further: ‘Have we implicitly used the normal distribution yet?'

Although we have proceeded no further than presenting the pooled estimate under the common‐effect model (standard errors, confidence intervals and so on are conspicuous by their absence), it is not entirely clear whether or not the normal distribution was used when presenting the common‐effect pooled estimate. The answer to our question is subtle, and depends upon the justification that was used to motivate this particular estimate. Ignoring, for now, the uncertainty in the within‐study variances $s_{i}^{2}$, and so the weights $w_{i}=s_{i}^{-2}$, the estimate $\hat{\mu }=\sum w_{i}Y_{i}/\sum w_{i}$ could be justified on the grounds that, if we are to estimate $\hat{\mu }$ using a linear combination of the $Y_{i}$, then the use of any other set of weights that provide an unbiased estimate would result in a pooled estimate of μ with greater variance under the common‐effect model. Although some very weak assumptions are required in this justification, such as assuming that the $Y_{i}$ have finite variance, this argument for presenting $\hat{\mu }=\sum w_{i}Y_{i}/\sum w_{i}$ does not require any particular distributional assumption. However, if we assume that the $\varepsilon_{i}$, and so the $Y_{i}$, are normally and independently distributed, we can then write the common‐effect model as $Y_{i}∼N\left(\mu ,\sigma_{i}^{2}\right)$. Then the estimate $\hat{\mu }=\sum w_{i}Y_{i}/\sum w_{i}$ can also be justified on the grounds that it is the maximum likelihood estimate. In the absence of normality, however, estimators that are not weighted means may have better properties.

Matters are even more subtle under the random‐effects model (Ades, Lu, & Higgins, 2005; DerSimonian & Laird, 1986, 2015; Higgins, Thompson, & Spiegelhalter, 2009; Riley, Higgins, & Deeks, 2011). This model is a generalisation of the common‐effect model that allows for between‐study heterogeneity in the true underlying study effects. For the moment avoiding making distributional assumptions, we can write the random effects model as $Y_{i}=\mu_{i}+\varepsilon_{i}$, where all $\mu_{i}$ and $\varepsilon_{i}$ are independent. Here, $\text{E}(\mu_{i})=\mu$ and $\text{Var}\left(\mu_{i}\right)=\tau^{2}$, where τ^2^ is the between‐study variance. If $\tau^{2}=0$, so that all $\mu_{i}=\mu$, the random effects model collapses to the common‐effect model. If we assume that both the $\mu_{i}$ and the $\varepsilon_{i}$ are normally distributed, we can then write the random‐effects model as $Y_{i}∼N\left(\mu ,\sigma_{i}^{2}+\tau^{2}\right)$. The normal random‐effects model is often motivated by the hierarchical framework $Y_{i}|\mu_{i}∼N\left(\mu_{i},\sigma_{i}^{2}\right)$ and $\mu_{i}∼N\left(\mu ,\tau^{2}\right)$, where we refer to these two distributions as the within‐study and the between‐study distributions, respectively. The standard approach for making approximate inferences for μ under the random effects model initially estimates τ^2^ and then treats this parameter as if fixed and known, so that $w_{i}$ in the calculation above then becomes the reciprocal of the total estimated (within‐study plus the between‐study) variances, $w_{i}^{*}=1/\left(s_{i}^{2}+{\hat{\tau }}^{2}\right)$. Questions relating to the use of the normal distribution are now more complicated because many estimators of τ^2^ are possible (Veroniki et al., 2016). Some, but not all, of these estimators assume that the $\mu_{i}$ and $\varepsilon_{i}$ are normally distributed. Hence, when presenting the random effects model's estimate of μ, the answer to the question ‘Have we used the normal distribution yet?’ depends on the type of estimation method used for τ^2^ *and* the justification for using the random effects weights $w_{i}^{*}$.

The main point from this introduction is that issues relating to the use, or avoidance, of the normal distribution in meta‐analysis are more immediate, and complicated, than is necessarily obvious. The overall aims of this paper are to explore how the normal distribution is used in meta‐analysis and to consider the case for using it less often. The rest of the paper is set out as follows. In Section 2, we present two contrasting real examples that will motivate our discussion further. In Section 3, we discuss the within‐study distributional assumptions in the conventional ‘two‐stage’ approach to meta‐analysis. In Section 4, we discuss the use of the normal distribution to describe the variation between studies and in Section 5, we discuss the use of this distribution when making statistical inferences. In Section 6, we summarise eight main assumptions that are made by conventional methods for meta‐analysis and we postulate a ‘hierarchy of sensitivity’ for a variety of forms of statistical inferences to normality assumptions. We examine the implications of alternative models, that make less use of the normal distribution, for our examples in Section 7. We conclude with some discussion in Section 8.

## 2 TWO REAL EXAMPLES

In this section, we present two contrasting real examples that will be used to exemplify the issues. The first of these involves individual‐level comparative binary outcome data, which can be presented as a series of 2 × 2 tables. The second example involves individual‐level continuous outcome data that is highly skew and where the individual patient data are available. In this section, we will perform standard ‘two‐stage’ analyses that meta‐analysts will be familiar with. See Section 5 for further details concerning how the calculations in the second stage are performed, where we also discuss how the normal distribution can be used to justify them. Later sections will also discuss the shortcomings of the conventional methods used in these two examples.

### 2.1 Example one: Aversive smoking for smoking cessation

Our first example is taken from the Cochrane Review *Aversive smoking for smoking cessation* (Hajek & Stead, 2001). Here, aversion therapy is intended to invoke an association between the stimulus of smoking with an unpleasant stimulus in order to encourage trial participants to abstain from smoking. We examine the first meta‐analysis from this review (Analysis 1.1). This compares the effectiveness of rapid smoking as an unpleasant stimulus with ‘attention placebo’ control, where the control is roughly matched for therapist contact (Hajek & Stead, 2001). The outcome of interest is the binary outcome ‘abstinence at long‐term follow‐up’ and the odds ratio was used to measure the treatment effect. This example involves 12 studies that include a total of 536 participants. The data exhibit little evidence of between‐study heterogeneity (the Cochrane Review reports a χ^2^ test statistic for heterogeneity of 6.87 on 11 degrees of freedom, and so a *P*‐value of 0.81, and $I^{2}=0$%). An alternative common‐effect Mantel–Haenszel method was used in the Cochrane Review to estimate a pooled odds ratio of 2.01 (with a 95% confidence interval from 1.36 to 2.95). An odds ratio that is greater than one indicates a treatment benefit and so we infer that rapid smoking is more effective for this outcome than the control.

Here, we re‐analyse these data using the random effects model and the conventional pooling method described in the introduction. We use the R package *metafor* (Viechtbauer, 2010) to perform two‐stage analyses of both example datasets. In the first stage, we calculate the study‐specific outcome data. Here, the $Y_{i}$ are the estimated log odds ratios. We used the *escalc* function from the *metafor* package to compute these estimates and their within‐study variances. One of the studies contains a zero count and we used the defaults of *escalc* to deal with this, and so to avoid infinite log odds ratios: halves were added to all counts in this particular study, but other studies were not modified in this way. Defining $A_{i}$ and $B_{i}$ to be the number of events (abstinence) and nonevents in the treatment group of the *i*‐th study, and $C_{i}$ and $D_{i}$ to be these same quantities in the corresponding control group, in the first stage *escalc* uses the standard formulae (Borenstein et al., 2009) to calculate the outcome data $Y_{i}=\log\left(\left(A_{i}/B_{i}\right)/\left(C_{i}/D_{i}\right)\right)$ and $s_{i}^{2}=1/A_{i}+1/B_{i}+1/C_{i}+1/D_{i}$.

In the second stage, we take these outcome data and perform the pooling. The restricted maximum likelihood (REML), the DerSimonian and Laird (1986) and the Paule and Mandel (1982) estimators all provide ${\hat{\tau }}^{2}=0$. Hence, all three of the resulting random effects meta‐analyses collapse to the same common‐effect analysis. This common‐effect analysis provides $\hat{\mu }=0.65$, where μ represents the population average log odds ratio, with standard error of 0.20. The corresponding 95% confidence interval is [0.25, 1.05] and the results for this example are summarised in Table 1. Transforming the estimate and confidence interval to the odds ratio scale gives a pooled odds‐ratio of 1.92 (with a 95% confidence interval from 1.29 to 2.85). These results are in broad agreement with those from the Cochrane Review.

**Table 1:** Example 1: Inferences for μ

Analysis	Estimate	95% Confidence interval
REML (Section 2.1)	0.65	[0.25, 1.05]
DL (Section 2.1)	0.65	[0.25, 1.05]
PM (Section 2.1)	0.65	[0.25, 1.05]
Logistic regression (Section 7)	0.71	[0.32, 1.10]

DL and PM indicate that the DerSimonian and Laird, and Paule Mandel estimators of τ^2^ have been used, respectively

**Figure 1:**
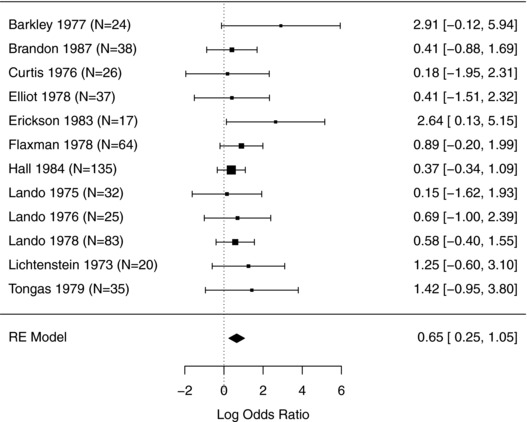
Forest plot for example one: Aversive smoking for smoking cessation. The results are presented as being from the random effects model, but this collapses to a common‐effect model for all three estimators of τ^2^. The number of participants in each study is indicated by *N*

Although all three point estimates of τ^2^ are zero, the uncertainty in this estimate is usually considerable in practice. A 95% confidence interval for τ^2^, using the *Q* profile method (Knapp, Biggerstaff, & Hartung, 2006; Viechtbauer, 2007), is [0, 0.90]. This confidence interval indicates that τ^2^ is quite imprecisely estimated. The point estimate of τ^2^ lies at the lower bound of the confidence interval because it is zero and negative values for this parameter are not allowed. The test for heterogeneity provides a *P*‐value of 0.81, which is in agreement with the Cochrane Review. A forest plot, on the log‐odds scale, is shown in Figure 1.

### 2.2 Example two: The association between smoking and C‐reactive protein level

Our second example is also related to smoking, but this time we are interested in how participants’ smoking status may influence their blood concentration of C‐reactive protein (CRP). These data were provided by the Emerging Risk Factors Collaboration (2007). In our dataset, we have individual participant data from 40 studies and a total of 170,201 participants. Our interest lies in the association between participants' smoking status and CRP level, adjusted for age and sex. In the first stage, we perform standard linear regressions to estimate study‐specific adjusted associations of smoking status with CRP level and so calculate the outcome data for the meta‐analysis. However, the participants' CRP levels were found to be highly skewed, and so it was not surprising that residual plots of linear regressions of CRP level on smoking status, age, and sex indicated very poor model fits. Hence, the CRP levels were log‐transformed prior to analysis and so we fitted 40 study‐specific linear models of the form:

(1) $$\log\left({\text{CRP}}_{j}\right)=\alpha_{0}+\alpha_{1}{\text{age}}_{j}+\alpha_{2}{\text{sex}}_{j}+\beta {\text{smoke}}_{j}+\varepsilon_{j}$$

for j=1,2,…n, where *n* is the number of participants in the study in question, age_*j*_ is the *j*‐th participant's age and sex_*j*_ and smoke_*j*_ are indicators for sex (1 for female; 0 for male) and current smoking status (1 for a current smoker; 0 otherwise). In this model, β is the parameter of primary interest, where exp(β) measures the proportional increase in CRP level that is associated with smoking, controlling for age and sex. In studies where all participants are the same sex, controlling for sex is inherent in the design and neither required nor possible in the analysis, so in these studies, the term $\alpha_{2}{\text{sex}}_{j}$ was omitted from model (1).

In this meta‐analysis, the 40 study‐specific estimates of β provide the outcome data $Y_{i}$ for the meta‐analysis, and their within‐study variances $s_{i}^{2}$ are obtained when fitting the standard linear regressions shown in (1). A common practical difficulty when fitting study‐specific regression models such as (1) occurs when studies collect different sets of covariates, but this is not an issue here.

**Figure 2:**
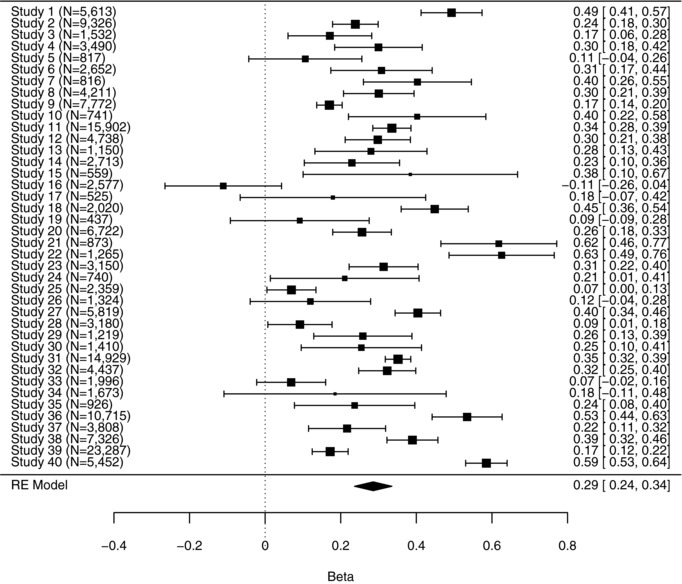
Forest plot for example two: The association between smoking and C‐reactive protein level. The results are from the random‐effects model where τ^2^ is estimated using REML. The numbers of participants in each study are indicated by *N*

In the second stage, these outcome data were pooled in random effects meta‐analyses. The REML, the DerSimonian and Laird (1986) and the Paule and Mandel (1982) estimators are very similar (${\hat{\tau }}^{2}=$0.021, 0.019 and 0.021 to three decimal places, respectively). The REML analysis provides $\hat{\mu }=0.29$, where μ represents the population average regression coefficient β, with standard error of 0.025. The corresponding 95% confidence interval is [0.24, 0.34]. Exponentiating this pooled estimate and its confidence interval, to make inferences about the pooled proportional increase in CRP level associated with smoking status, provides a point estimate for expμ of 1.33 with 95% confidence interval (1.27, 1.40) and the results for this example are summarised in Table 2. This analysis suggests that, on average, smoking is associated with an increase in CRP level of about one third. A 95% confidence interval for τ^2^, using the Q profile method (Knapp et al., 2006; Viechtbauer, 2007), is [0.013, 0.037]. The test for heterogeneity provides a *P*‐value of less than 0.0001, which indicates that there is strong evidence of between‐study heterogeneity. This is reflected in the $I^{2}=93$% statistic reported by *metafor* in the REML analysis. A forest plot from the REML analysis is shown in Figure 2.

**Table 2:** Example 2: Inferences for μ

Analysis	Estimate	95% Confidence interval
REML (Section 2.2)	0.29	[0.24, 0.34]
DL (Section 2.2)	0.29	[0.24, 0.33]
PM (Section 2.2)	0.29	[0.24, 0.34]
T distribution (Section 7)	0.29	[0.24, 0.34]
Mixture distribution (Section 7)	0.29	[0.23, 0.33]

DL and PM indicate that the DerSimonian and Laird and Paule Mandel estimators of τ^2^ have been used, respectively

These two examples will serve to illustrate a wide variety of issues that provide the focus of the rest of the paper. We will begin by discussing the within‐study distributional assumptions. Although the same types of conventional within‐study assumptions were made when analysing both examples, our concerns about these assumptions are different in these two applications because of their contrasting nature.

## 3 WITHIN‐STUDY DISTRIBUTIONAL ASSUMPTIONS

As illustrated by our two examples above, in the first stage of conventional meta‐analyses we compute $Y_{i}$ and their within‐study variances $s_{i}^{2}$. In this section, we will discuss the implications of making within‐study distributional assumptions, that is, assumptions about the distributions $Y_{i}|\mu_{i}$, and we will also discuss ways to avoid these assumptions. From the description of the random‐effects model in the introduction, if we refrain from assuming within‐study normality under this model, we can write this conditional distribution as $Y_{i}=\mu_{i}+\varepsilon_{i}$, where $\mu_{i}$ is treated as fixed, $\text{E}(\varepsilon_{i})=0$ and $\text{Var}\left(\varepsilon_{i}\right)=\sigma_{i}^{2}$; otherwise the distributional form of $\varepsilon_{i}$ is unspecified. If we further assume within‐study normality, then we have $\varepsilon_{i}∼N\left(0,\sigma_{i}^{2}\right)$, so that $Y_{i}|\mu_{i}∼N\left(\mu_{i},\sigma_{i}^{2}\right)$. Under the common‐effect model, we have the stronger statements that $Y_{i}=\mu +\varepsilon_{i}$ and $Y_{i}∼N\left(\mu ,\sigma_{i}^{2}\right)$.

Within‐study assumptions are therefore similar under both the common‐effect and the random‐effects models, the only difference being whether or not we assume that all $\mu_{i}$ are the same. Approximating the $\sigma_{i}^{2}$ with the within‐study variances, $s_{i}^{2}$ is standard practice in both common‐effect and random‐effects meta‐analysis. However, we will see below that ignoring the uncertainty in the $s_{i}^{2}$ can have serious implications for the accuracy of the resulting statistical inference. The within‐study assumptions made in conventional meta‐analysis make three ‘hidden assumptions’. The first two of these hidden assumptions are not intrinsically related to within‐study normality, but all three assumptions are implied by the conventional within‐study approximations used in the analyses in Section 2.

### 3.1 Hidden assumption one: The estimates are unbiased

Perhaps the most basic assumption is that every $Y_{i}$ provides an unbiased estimate of the corresponding $\mu_{i}$. This is because our assumptions imply $\text{E}\left(Y_{i}|\mu_{i}\right)=\mu_{i}$, where $\mu_{i}=\mu$ in the common‐effect model. Even in the already idealised situation where publication biases or other types of internal biases are assumed to be absent (these types of bias are beyond the scope of this paper), this assumption is often patently false. For example, in Section 2.1 we are likely to be willing to assume that the studies provide approximately unbiased estimates of the probabilities of an event in the two treatment groups. However, even then the $Y_{i}$ will be biased because of what we will refer to as ‘transformation bias’. This is because the logit transformation is non‐linear. This type of bias is completely ignored in the analysis presented in Section 2.1 and may be serious in small studies.

### 3.2 Hidden assumption two: The within‐study variances are known

As explained above, standard methodologies ignore the uncertainty in the within‐study variances and so take $\sigma_{i}^{2}$ to be $s_{i}^{2}$ when modelling $Y_{i}|\mu_{i}$. This approximation is acceptable in large studies and we would hope that it is generally appreciated that this approximation is used. It is also worth noting that for most outcomes, the formulae for the within‐study variances are themselves merely an approximation, for example the within‐study variances of the log odds ratios in our first example are based on a first‐order Taylor series expansion and hence are asymptotically correct.

Jackson (2008) discusses the formal justification for approximating the $\sigma_{i}^{2}$ with their estimates $s_{i}^{2}$ when using normal within‐study approximations. Briefly, assuming that the studies are sufficiently large, the central limit theorem (CLT) is used to justify the use of the normal distributions and then a further approximation is used to take the variances as known.

There is also a more subtle hidden assumption made in conventional meta‐analysis methodologies: the correlation between the $Y_{i}$ and their within‐study variances $s_{i}^{2}$ is ignored. This is because, as explained above, the uncertainty in the $s_{i}^{2}$ is completely ignored. For some forms of outcome data, such as an unadjusted sample mean, *under the assumption that the raw data are normally distributed*, this association can be safely neglected. However, this type of assumption will only ever be approximately true in practice. This concern is potentially serious in our first example in Section 2.1 because the studies are small and the $Y_{i}$ and $s_{i}^{2}$ are correlated because they are calculated from the same data and there is no statistical theory that ensures their independence. For example, from the formulae $Y_{i}=\log\left(\left(A_{i}/B_{i}\right)/\left(C_{i}/D_{i}\right)\right)$ and $s_{i}^{2}=1/A_{i}+1/B_{i}+1/C_{i}+1/D_{i}$, an unusually low value of $B_{i}$ yields large values of both $Y_{i}$ and $s_{i}^{2}$.

### 3.3 Hidden assumption three: The shape of the normal distribution is assumed, not just the first two moments

A further hidden assumption is that, when assuming within‐study normality, we further make a statement about the shape of the within‐study distribution. For example, the common‐effect pooled estimate is the maximum likelihood estimate as a direct consequence of the normality assumptions, and other maximum likelihood estimates would in general be obtained if different distributional assumptions were made. Our first example raises obvious concerns about this assumption, because the studies are too small for the within‐study normal approximations to be anything other than crude. A further consequence of assuming within‐study normality is that we can then justify presenting the study‐specific confidence intervals shown in Figures 1 and 2.

The use of the $s_{i}^{2}$ as if they are the $\sigma_{i}^{2}$ (hidden assumption two) raises concerns about this final hidden assumption. This is because if we assume $Y_{i}|\mu_{i}∼N\left(\mu_{i},\sigma_{i}^{2}\right)$, as in both the common‐effect and random‐effects models, then we do not have $Y_{i}|\mu_{i}∼N\left(\mu_{i},s_{i}^{2}\right)$; even in the simplest possible situation where the $Y_{i}$ are sample means of normally distributed observations then, from standard textbook theory, the *t* distribution is required to make inferences for $\mu_{i}$ in situations where the population variance is unknown. However, we suggest that in practice the assumptions $Y_{i}|\mu_{i}∼N\left(\mu_{i},\sigma_{i}^{2}\right)$ and $Y_{i}|\mu_{i}∼N\left(\mu_{i},s_{i}^{2}\right)$ should be both regarded as statistical approximations, where the second assumption is slightly cruder than the first. If the $\sigma_{i}^{2}$ were truly known, then the second hidden assumption would be true but standard methods could still be criticised because of concerns relating to the other two hidden assumptions.

The widespread use of within‐study approximations is perhaps one of the biggest concerns about the current standard practice in meta‐analysis. See Stijnen, Hamza, and Özdemir (2010) for a good discussion of why approximate within‐study normal approximations ‘might not always be appropriate’. Critiques of the within‐study modelling in conventional two‐stage meta‐analyses can be found. Hoaglin (2015) argues that this is an ‘incorrect (but convenient) assumption’. Shuster and Walker (2016) more directly identify one of the concerns that we have described and they state that the ‘variance estimate for an individual‐study‐level log of the relative risk is associated with the direction of the sampling error, inducing bias’.

### 3.4 Methods that explicitly address the hidden assumptions

There have been various attempts to ‘fix‐up’ particular aspects of the two‐stage approach by better allowing for the nature of the $Y_{i}$ and their within‐study variances $s_{i}^{2}$. For example, Chang, Waternaux, and Lipsitz (2001) and Emerson, Hoaglin, and Mosteller (1993) consider using weighted average proportions of events, rather than study‐specific proportions, when computing the within‐study variances. This modification of the conventional methodology directly addresses the concern that the $Y_{i}$ and $s_{i}^{2}$ are correlated when the individual‐level data are binary (see Section 3.2).

Böhning et al. (2002) and Malzahn, Böhning, and Holling (2000) develop methodologies for estimating the between‐study variance that acknowledge that the within‐study variances depend on unknown parameters. This type of methodology is therefore directly motivated by the second hidden assumption (Section 3.2). Kulinskaya, Dollinger, and Bjørkestøl (2011a, 2011b); Kulinskaya and Dollinger (2015) develop methods for testing for the presence of heterogeneity (see also Section 5) that avoid making idealised assumptions about the $Y_{i}$ and $s_{i}^{2}$. Briefly, Kulinskaya et al. (2011a, 2011b); Kulinskaya and Dollinger (2015) use gamma approximations for the distribution of the conventional *Q* statistic (see Section 5.3), under the null hypothesis that $\tau^{2}=0$, where the parameters of this gamma approximation are calculated using more accurate and realistic distributions. These methods are therefore motivated by all three hidden assumptions (Sections 3.1–3.3). A closely related idea is to use non‐normal within‐study distributions that better describe the nature of the study outcome data in likelihood‐based analyses. Iyengar and Greenhouse (1988) assume within‐study *t* distributions in their two‐stage common‐effect meta‐analyses (and include models for publication bias but do not allow for between‐study heterogeneity).

Stijnen et al. (2010) suggest using the non‐central hypergeometric distribution in one‐stage random‐effects analyses, to describe two by two tables where the odds ratio is the outcome measure used, as in our first example. These methods explicitly avoid using the normal distribution and so are perhaps most directly motivated by the third hidden assumption (Section 3.3), but these methods also address the other two hidden assumptions. Generalised linear mixed models (GLMMs), that facilitate a one‐stage approach (Böhning, Mylona, & Kimber, 2015; Simmonds & Higgins, 2016; Stijnen, Hamza, and Özdemir, 2010), appear to be the primary proposal for avoiding within‐study approximations when performing random‐effects meta‐analyses. Generalised linear models (such as logistic regressions) can be used to fit the corresponding common‐effect analyses. However, the most appropriate types of GLMM to use in applied work remains an open question. For example, for comparative binary outcome data (as in our first example in Section 2.1), Simmonds and Higgins (2016) suggest fitting the GLMM

(2) $$g\left(\pi_{ij}\right)=\phi_{i}+\mu_{i}x_{ij},$$

where $\pi_{ij}$ is the probability of an event in the *j*‐th treatment group (j=1: treatment; j=0 control) in the *i*‐th study, $\phi_{i}$ is the baseline risk of the event in the *i*‐th study, $\mu_{i}∼N\left(\mu ,\tau^{2}\right)$, $x_{ij}$ is an indicator for the treatment group and g(·) is the link function. For example, by taking g(·) to be the logit function, μ and τ^2^ then represent the average log‐odds ratio and the corresponding between‐study variance, so that these two parameters represent the same quantities estimated in Section 2.1 using more conventional methodology. An issue with model (2) is that, because there is a separate fixed effect $\phi_{i}$ for every study, the number of parameters increases at the same rate as the number of studies. Hence, the usual asymptotic theory of maximum likelihood does not apply (Jackson, Law, Stijnen, Viechtbauer, & White, 2018). One way to avoid this difficulty is to assume that $\phi_{i}∼N\left(\phi ,\sigma^{2}\right)$, but this is equivalent to a reduced form of the joint bivariate model for the control and treatment event probabilities (van Houwelingen, Arends, & Stijnen, 2002), which allows the recovery of inter‐trial information (Senn, 2010) which can lead to bias (Jackson et al., 2018). See Jackson et al. (2018) for some examples with substantial between‐study heterogeneity that illustrate the options available to the analyst.

### 3.5 Current practice

Although methodologies that avoid using normal approximations within studies have been proposed, these more sophisticated methods are rarely seen in application. There are however two main exceptions to this. Firstly, individual patient data meta‐analyses can be performed as both one‐ and two‐stage analyses, where one‐stage analyses avoid the use of within‐study approximations. It is perhaps for this reason that one‐stage analyses of Individual Patient Data (IPD) are frequently used, although other motivations include the desire to examine covariate effects and non‐linear associations. One‐stage meta‐analyses of IPD have been suggested for a variety of outcomes (e.g. Tudur Smith, Williamson, & Marson, 2005; Turner, Omar, Yang, Goldstein, & Thompson, 2000; Whitehead et al., 2001). Section 2.2 provides an example of a two‐stage IPD meta‐analysis.

Secondly, the analysis of diagnostic test studies is often performed using the bivariate framework described by Harbord and Whiting (2009). In its simplest form, this methodology uses intercept only logistic regressions for the within‐study models (and so uses a GLMM) that avoid the use of within‐study approximations. This methodology has proved popular because diagnostic test studies are often small and the sensitivities and specificities that are described by this type of model are often close to one, so that within‐study normal approximations would then be especially crude.

These two types of application provide concrete examples of the fact that meta‐analysts are willing to adopt methods that avoid the use of within‐study normal approximations. Random‐effects implementations further require distributional assumptions between studies, and we discuss this issue next.

## 4 BETWEEN‐STUDY DISTRIBUTIONAL ASSUMPTIONS

We now turn our attention to distributional assumptions made between studies, that is the distribution of the $\mu_{i}$. The situation is trivial, and so made entirely clear, under the common‐effect model. This model makes the strong, and usually difficult to defend, assumption that all $\mu_{i}=\mu$. Hence, we will restrict our discussion to random‐effects models.

As explained above, whilst refraining from making a distributional assumption, we assume that $\text{E}(\mu_{i})=\mu$ and $\text{Var}\left(\mu_{i}\right)=\tau^{2}$. Upon further assuming between‐study normality, we have $\mu_{i}∼N\left(\mu ,\tau^{2}\right)$. Hence important, and perhaps sometimes overlooked, between‐study distributional assumptions are made in random‐effects meta‐analyses. Even in situations where between‐study normality assumptions can be avoided (e.g. as Higgins et al. (2009) point out, the usual confidence interval for μ from the DerSimonian and Laird, 1986, method ‘will be valid approximately in a distribution‐free context when there are many studies'), the assumption that all $\mu_{i}$ share a common mean is required. However, many implementations of the random‐effects model further require the assumption that the $\mu_{i}$ are normally distributed, for example estimators of τ^2^ that make this assumption may be used. In many respects, the implications of the conventional between‐study distributional assumption, $\mu_{i}∼N\left(\mu ,\tau^{2}\right)$, are similar to those discussed in the context of the three hidden within‐study assumptions. For example, we assume that $\text{E}(\mu_{i})=\mu$ to avoid bias (see also Section 3.1) and we assume the shape of the normal distribution (see also Section 3.3).

The issues surrounding between‐study normality assumptions are however somewhat different to those within‐studies. This is emphasised by Baker and Jackson (2008) who note that, the CLT ‘does not really imply anything for the distribution of the random effect. We can only appeal to the CLT here with the vague idea that the unknown source of variation between studies might be the sum of several factors’. Hardy and Thompson (1998) suggest some practical strategies for assessing this assumption, but to perform well these methods require more studies than are usually available. These strategies include informal inspections of normal probability plots and more formal hypothesis testing procedures.

### 4.1 Methods that explicitly avoid making between‐study normality assumptions

Likelihood based methods (including Bayesian analyses) that assume non‐normal random‐effects distributions have been suggested (Baker and Jackson, 2008; Baker & Jackson, 2016; Beath, 2014; Lee & Thompson, 2008). These alternative random‐effects distributions are usually motivated by the presence of outliers or other unusual sets of study results. When outliers are present, skew or heavy tailed random‐effects distributions down‐weight them and can produce results that are more robust. However, a difficulty is that sophisticated models for the random‐effects are hard to identify. A related idea is presented by Gumedze and Jackson (2011), who remain in the framework of assuming normally distributed random‐effects, but allow some observations to possess more variance so that they are down‐weighted.

Our second example raises some legitimate concerns about the between‐study distributional assumptions made in conventional random‐effects meta‐analyses. Here there is considerable between‐study heterogeneity, where from Figure 2 it is evident that studies in this population provide markedly different findings. There would therefore seem to be some potential for alternative random‐effects distributions to provide substantively different conclusions.

To summarise this discussion, the between‐study normality assumption has occasionally been challenged and alternative random‐effects distributions have been proposed. However, this issue has not received as much attention as normality assumptions within‐studies has (see Section 3).

## 5 NORMAL ASSUMPTIONS WHEN MAKING INFERENCES

The previous two sections have addressed the assumptions made in the statistical modelling. We will now focus on the use of the normal distribution when making inferences.

### 5.1 Inferences for μ

When discussing the implications of the conventional within‐study distributional assumptions, we focused on bias (Section 3.1), treating the within‐study variances as known (Section 3.2) and the shape of the normal distribution (Section 3.3). The implications of the distributional assumptions for $\hat{\mu }$ when making inferences for μ are similar but these three assumptions now apply to the pooled effect. As in Section 3, we will examine each of these assumptions in turn. The hidden within‐study assumptions described in Sections 3.1–3.3 all impact on the plausibility of the hidden assumptions for $\hat{\mu }$ that follow: if the hidden within‐study assumptions described above are correct or, more realistically, are approximately true then the hidden assumptions for $\hat{\mu }$ are more likely to be reasonable.

#### 5.1.1 Hidden assumption one: The pooled estimate is unbiased

We have already explained that the pooled estimates are $\hat{\mu }=\sum w_{i}Y_{i}/\sum w_{i}$ and $\hat{\mu }=\sum w_{i}^{*}Y_{i}/\sum w_{i}^{*}$ under the common‐effect and random‐effects models, respectively. Conventional methods assume that these pooled estimates are unbiased. This assumption will raise concerns in situations where the $Y_{i}$ are biased (see Section 3.1) and/or where the $Y_{i}$ and $s_{i}^{2}$ are correlated (see Section 3.2). Correlation between the outcome data $Y_{i}$ and the within‐study variances $s_{i}^{2}$ is a particular source of concern under the common‐effect model because it will directly cause correlation between $Y_{i}$ and the study weights $w_{i}$. This correlation could result in notable bias in $\hat{\mu }$, even in situations where the $Y_{i}$ are themselves unbiased. Correlation between the $Y_{i}$ and the study weights $w_{i}^{*}$ is also a concern under the random‐effects model. However as ${\hat{\tau }}^{2}$ becomes larger, the weights $w_{i}^{*}$ become more similar, so that any association between the $Y_{i}$ and $w_{i}^{*}$ becomes increasingly diluted. However, the assumption that the pooled estimate is unbiased is a potential cause for concern in all conventional meta‐analyses.

#### 5.1.2 Hidden assumption two: The variance of the pooled estimate is known

Standard methods for common‐effect and random‐effects meta‐analysis assume that the variance of the pooled estimates is known. This is because the conventional methodologies ignore the uncertainty in the $s_{i}^{2}$ and random‐effects analyses further ignore the uncertainty in ${\hat{\tau }}^{2}$. Hence in the second stage of analysis when pooling the $Y_{i}$, we approximate $\sigma_{i}^{2}$ with $s_{i}^{2}$ and, in random‐effects meta‐analyses, τ^2^ with ${\hat{\tau }}^{2}$. These approximations greatly simplify the analysis: the standard error of $\hat{\mu }$ can then be shown to be $\text{SE}\left(\hat{\mu }\right)=1/\sqrt{\sum w_{i}}$ and $\text{SE}\left(\hat{\mu }\right)=1/\sqrt{\sum w_{i}^{*}}$ under the common‐effect and random‐effects models, respectively. These approximate standard errors can be justified without resorting to using normal distributions provided that, under the random‐effects model, estimates of τ^2^ are not motivated in this way. However, these standard errors are not truly known, and the accuracy of the statistical approximations that take them to be known depends on the precision of the estimated variance components that are used to calculate them. For accurate inference, we therefore require reasonably large studies so that the $s_{i}^{2}$ are precisely estimated in both common‐effect and random‐effects meta‐analyses. In random‐effects meta‐analyses, we also require a reasonably large number of studies in order to estimate τ^2^ with acceptable precision. In practice, the number of studies is often small and so this hidden assumption is likely to be a greater concern in random‐effects meta‐analyses.

#### 5.1.3 Hidden assumption three: The shape of the normal distribution is assumed for the pooled estimate, not just the first two moments

If we make all three hidden assumptions, then confidence intervals and hypothesis tests for μ immediately follow from very simple calculations using the properties of the normal distribution. Defining *Z* to be an appropriate critical value of the standard normal distribution, we calculate confidence intervals as $\hat{\mu }\pm Z\times \text{SE}\left(\hat{\mu }\right)$. In Section 2, we took Z=1.96 to compute 95% confidence intervals. The reader may note that this is the first time that we have not allowed any room for avoiding a statement that involves the normal distribution when using conventional methods.

In order to attempt to account for the uncertainty in τ^2^ in random‐effects meta‐analyses, Higgins et al. (2009) argue that a ‘*t* distribution should provide a better basis than a normal distribution’. However, as they point out, determining effective degrees of freedom is difficult. This is because, the random‐effects model deviates from the usual textbook situations where the use of the *t* distribution can be properly justified. Furthermore, this standard theory requires normality assumptions and so we suspect that any rigorous justification of the use of the *t* distribution for this purpose is likely to make some form of normality assumption for $\hat{\mu }$.

Although the implications of all three hidden assumptions for $\hat{\mu }$ are a potential cause of concern when performing common‐effect and random‐effects meta‐analyses, the use of the normal distribution when calculating confidence intervals and performing hypothesis tests under the random‐effects model has received considerable attention. We therefore address this issue in the section immediately below. The concerns that we will describe when calculating confidence intervals also apply when performing hypothesis tests. For example, actual coverage probabilities of 95% confidence intervals that are less than the nominal level also manifest themselves as hypothesis tests at the 5% significance level that are anti‐conservative.

### 5.2 Concerns about the use of the normal distribution when calculating confidence intervals for μ

Matters are simple under the common‐effect model, where normal within‐study distributional assumptions imply that $\hat{\mu }$ is also normally distributed. However, the standard result that justifies this (that a linear combination of independent normals is also normally distributed) requires that the coefficients in the linear combination are constants, whereas the common‐effect weights, $w_{i}=s_{i}^{-2}$, are estimates. This is ignored in the conventional modelling (see Section 3). Hence, even under the common‐effect model, the assumption that $\hat{\mu }$ is normally distributed is more objectionable than is necessarily immediately obvious. Hence, there is the concern that confidence intervals may also be inaccurate under the common‐effect model.

Many concerns have been expressed that relate directly to the accuracy of the usual random‐effects approach for calculating confidence intervals for μ and/or the corresponding hypothesis tests (e.g. Brockwell & Gordon, 2001; Follmann & Proschan, 1999; Guolo & Varin, 2017; IntHout, Ioannidis, & Borm, 2014). This has resulted in a variety of alternative methodologies (e.g. Bellio & Guolo, 2016; Biggerstaff & Tweedie, 1997; Böhning et al., 2002; Guolo, 2012; Hardy & Thompson, 1996; Hartung, 1999; Hartung & Knapp, 2001a, and Hartung & Knapp, 2001b; Malzahn et al., 2000; Noma, 2011; Sidik & Jonkman, 2002). Of these suggestions, the Hartung and Knapp modification, which was also independently suggested by Sidik and Jonkman, is probably the best known and simplest idea, and this particular method has been advocated for widespread use (IntHout et al., 2014). However, concerns about this alternative methodology have also been raised (Jackson, Law, Rücker, & Schwarzer, 2017; Wiksten, Rücker, & Schwarzer 2016), mainly on the grounds that this modification can result in analyses that are not conservative compared to a common‐effect analysis. The main source of concern about using the usual random‐effects methodology appears to be that there are often too few studies to estimate τ^2^ with reasonable precision. Bayesian analyses with informative priors (e.g. Pullenayegum, 2011; Rhodes, Turner, & Higgins, 2015) have been proposed as a way of resolving this difficulty, but come at the price of making additional assumptions via these evidence‐based priors.

In order to analytically explore the accuracy of conventional confidence intervals for μ under the random‐effects model, Jackson and Bowden (2009) derive the distribution of a standardised version of $\hat{\mu }$ under the very idealised setting where all within‐study variances are not only known exactly, but are also identical. By deriving a distribution of $\hat{\mu }$ under the random‐effects model, their investigation acknowledges that $\hat{\mu }$ is not truly normally distributed with known variance. This is clearly an artificial scenario, but it is sufficient to show that the conventional normal approximation for $\hat{\mu }$ under this model is not very accurate unless the number of studies is large (Jackson and Bowden suggest that ten studies is adequate). Jackson and Bowden (2009) assume that the DerSimonian and Laird (1986) estimator of τ^2^ is used in their analysis, but Jackson et al. (2017) have subsequently shown that this estimator is equivalent to both the REML and Paule‐Mandel estimators in this setting. Zeng and Lin (2015) also assume that the DerSimonian and Laird estimator is used and established, for finite *k*, that $\hat{\mu }$ does not tend towards normality under the random‐effects model as the study sizes become large. One reason for assuming normality within (see Section 3) and between (see Section 4) studies might be to justify using the usual normal approximation for $\hat{\mu }$ with small *k*, with the intuition that we are then likely to require fewer studies to assume that $\hat{\mu }$ is approximately normally distributed. However, the analyses presented by Jackson and Bowden (2009) and Zeng and Lin (2015) are sufficient to establish that, even in idealised situations where the random‐effects model is true, we require sufficiently large numbers of studies to take $\hat{\mu }$ to be approximately normally distributed under the random‐effects model.

### 5.3 Inferences for τ^2^

Point estimates of τ^2^ can be obtained under the random‐effects model using a variety of estimators and three of these were used in Section 2. See Veroniki et al. (2016) for full details of these estimators that can be broadly split into two categories: moment based or likelihood based (where the Bayesian approaches are placed in the second category). Subject to the issues and concerns raised about the within‐study modelling described in the previous section, moment‐based estimators of τ^2^ are valid without the necessity to make normality assumptions. However, the moment‐based estimators possess no optimality properties and all the estimators described by Veroniki et al. (2016) make the first and second hidden assumptions (Sections 3.1 and 3.2). By assuming within‐ and between‐study normality, the more statistically principled, and in some senses optimal, likelihood‐based methods described by Veroniki et al. (2016) can then be properly justified. Returning to the analyses in Section 2, this means that, strictly speaking, the REML analyses required normality assumptions both within‐ and between‐studies, whereas the DerSimonian and Laird (1986) and the Paule and Mandel (1982) analyses did not. However Kontopantelis and Reeves (2012a, 2012b) show that likelihood‐based analyses are robust to departures from normality. Hence, the REML analysis could be justified on the grounds that it is the preferred estimator of τ^2^ if the normality assumptions are true, and is also approximately valid if these assumptions are false.

Further inferences for τ^2^ were also provided in Section 2, specifically hypothesis tests for the presence of heterogeneity were performed, confidence interval for τ^2^ were computed and *I* ^2^ statistics were quoted. All of these inferences can be derived from ‘*Q* statistics or pivots’. We define

(3) $$Q\left(\tau^{2}\right)=\sum w_{i}\left(\tau^{2}\right){\left(Y_{i}-\hat{\mu }\left(\tau^{2}\right)\right)}^{2},$$

where $w_{i}\left(\tau^{2}\right)=1/\left(s_{i}^{2}+\tau^{2}\right)$ and $\hat{\mu }\left(\tau^{2}\right)=\sum w_{i}\left(\tau^{2}\right)Y_{i}/\sum w_{i}\left(\tau^{2}\right)$; this notation emphasises the dependence of the calculations on τ^2^. The conventional *Q* statistic is then given as Q=Q(0). The standard test for heterogeneity computes *Q* and compares this to a critical value of a $\chi_{k-1}^{2}$ distribution; if *Q* is large in relation to the $\chi_{k-1}^{2}$ distribution, then the test is taken to mean that there is evidence of between‐study heterogeneity. This test requires the usual within‐study normality assumptions and so all three hidden assumptions in Sections 3.1–3.3 are a cause of concern when performing this test. Confidence intervals for τ^2^ from the *Q* profile method (Knapp et al., 2006; Viechtbauer, 2007) contain all values of τ^2^ such that $Q(\tau^{2})$ lies between critical values from the $\chi_{k-1}^{2}$ distribution. In Section 2, equal‐tailed 95% confidence intervals for τ^2^ were provided, so that the 0.025 and 0.975 quantiles were used. If no τ^2^ provides $Q(\tau^{2})$ in this range, then this is because the data are very homogeneous (Knapp et al., 2006; Viechtbauer, 2007) and either a null confidence set or the interval (0,0)={0} is usually given. This is because the study results are even more homogeneous than expected under the assumption that $\tau^{2}=0$.

Finally, the *I* ^2^ statistic (Higgins and Thompson, 2002), loosely speaking, describes the proportion of the variation in the outcome data that is attributed to between‐study heterogeneity. This statistic can be expressed as $I^{2}={\hat{\tau }}^{2}/\left(s^{2}+{\hat{\tau }}^{2}\right)$, and expressed as a percentage, where *s* ^2^ is a ‘typical’ or ‘representative’ within‐study variance (Higgins and Thompson, 2002). If the DerSimonian and Laird estimator of τ^2^ is used, then we have $I^{2}=\left(Q-\left(k-1\right)\right)/Q$, where negative *I* ^2^ are truncated to zero. The *I* ^2^ statistic is also subject to some of the issues that we discuss, in particular the within‐study variances are used in computation and taken as fixed and known when interpreting the magnitude of this statistic. However, the *I* ^2^ statistic does not introduce any new normality assumptions.

These methods for making inferences for τ^2^ under the random‐effects model have been criticised on the grounds that the assumptions required by them may not be sufficiently accurate. Hoaglin (2016a) and Kulinskaya et al. (2011a, 2011b); Kulinskaya and Dollinger (2015) clarify that the usual distributional assumptions for the *Q* statistic described above rely on idealised normality assumptions where, in particular, the within‐study distributions are treated as if known. As Hoaglin (2016a, 2017) point out, the approximate nature of our distributional assumptions when computing *Q* can also have unfortunate implications for the DerSimonian and Laird (1986) estimator and the *I* ^2^ statistic. The *Q* profile method used for our examples requires the assumption that $Y_{i}∼N\left(\mu ,\sigma_{i}^{2}+\tau^{2}\right)$, where the $\sigma_{i}^{2}$ are approximated by their estimates $s_{i}^{2}$. This is so that, from equation (3), the distributional assumption $Q\left(\tau^{2}\right)∼\chi_{k-1}^{2}$ is correct. These comments also apply to closely related methods based on alternative *Q* statistics (Jackson, 2013; Jackson, Turner, Rhodes, & Viechtbauer, 2014) and also an approximate version of this methodology (Jackson, Bowden, & Baker, 2015) that has been criticised for this and other reasons (Hoaglin, 2016b). Likelihood‐based methods for computing confidence intervals for τ^2^ (Biggerstaff & Tweedie, 1997) also make these assumptions. The overall impression therefore is that methods for making further inferences for the magnitude of the between‐study variance, such as computing *I* ^2^ statistics and confidence intervals for τ^2^, can be anticipated to be especially sensitive to departures from the assumptions typically made in meta‐analyses.

### 5.4 The prediction interval for the true effect in a new study

A further type of statistical inference, that has become advocated for routine use and so we include it in our discussion, is a prediction interval for the true effect in a new study, μ_new_, from a random‐effects meta‐analysis. Higgins et al. (2009) and Riley et al. (2011) suggest the prediction interval

(4) $$\hat{\mu }\pm t_{k-2}\sqrt{{\hat{\tau }}^{2}+\text{SE}{\left(\hat{\mu }\right)}^{2}},$$

where $\hat{\mu }$ is the estimate under the random‐effects model, $\text{SE}(\hat{\mu })$ is the corresponding standard error under this model and $t_{k-2}$ is a critical value from a *t* distribution with (k−2) degrees of freedom; for a 95% prediction, interval $t_{k-2}$ is taken to be the 0.975 quantile.

If τ^2^ and the $s_{i}^{2}$ are treated as known, then the prediction interval in (4) can be motivated by assuming that $\mu_{\text{new}}-\hat{\mu }∼N\left(0,\tau^{2}+\text{SE}{\left(\hat{\mu }\right)}^{2}\right)$, where $\hat{\mu }$ and $\text{SE}(\hat{\mu })$ are calculated under the random‐effects model using weights of $1/(s_{i}^{2}+\tau^{2})$. As explained by Higgins et al. (2009), this follows from assuming that $\mu_{\text{new}}∼N\left(\mu ,\tau^{2}\right)$, where μ_new_ is independent of $\hat{\mu }$. Under these assumptions, the appropriate prediction interval is therefore $\hat{\mu }\pm Z\sqrt{\tau^{2}+\text{SE}{\left(\hat{\mu }\right)}^{2}}$. Higgins et al. (2009) and Riley et al. (2011) proposed the ad hoc modification of using a $t_{k-2}$ distribution to allow for the uncertainty in ${\hat{\tau }}^{2}$, which gives rise to (4). This prediction interval is therefore motivated by normality assumptions, but is not fully justified by them. Partlett and Riley (2017) show that the prediction interval (4) has some poor properties even when the random‐effects model is true. More pertinent to our discussion is that Lee and Thompson (2008) conclude that predictive distributions are sensitive to the distributional assumptions for the random effects. We can therefore anticipate that criticisms of the prediction interval (4), on the grounds that it is sensitive to the normality assumptions that motivate it, are likely to arise in the future.

The key additional distributional assumption required by the prediction interval in (4) is $\mu_{\text{new}}∼N\left(\mu ,\tau^{2}\right)$. This assumption relates to the true effect in a hypothetical new study and so is not testable. Some form of distributional assumption for μ_new_ is needed to compute a prediction interval and it is, at best, very difficult to motivate the use of any other distribution for this purpose. Despite this, meta‐analysts should be clear that this additional assumption is made when computing prediction intervals.

## 6 A SUMMARY OF EIGHT MAIN ASSUMPTIONS MADE BY CONVENTIONAL METHODS FOR META‐ANALYSIS

Our discussion has identified eight main assumptions that are made by conventional methods for meta‐analysis. We summarise these assumptions in Table 3. Here, we focus on the assumptions required to make inferences about the average effect (including the prediction interval for the true effect in a new study) rather than those required to make further inferences for τ^2^. This is because, the inferences for μ are of primary interest and the types of further inferences for τ^2^ described in Section 5.3 are often not used in application.

**Table 3:** Eight main assumptions made by conventional methods for meta‐analysis

Assumption	Most serious implication for μ if false	Especially dubious when
1. $Y_{i}$ unbiased for $\mu_{i}$ (Section 3.1)	Biased pooled estimate	Sparse non‐continuous data
2. Variances $s_{i}^{2}$ known (Section 3.2)	Inaccurate variance for $\hat{\mu }$	Small studies, sparse or skew data
3. $Y_{i}|\mu_{i}$ normal (Section 3.3)	Inaccurate likelihood‐based inference	Small studies, sparse or skew data
4. $\mu_{i}$ normal (Section 4)	Inaccurate likelihood‐based inference	Outlying studies are present
5. $\hat{\mu }$ unbiased for μ (Section 5.1.1)	Biased pooled estimate	$Y_{i}$ biased for $\mu_{i}$
6. Variance of $\hat{\mu }$ known (Section 5.1.2)	Inaccurate confidence interval	Few studies present; imprecise $s_{i}^{2}$
7. $\hat{\mu }$ normal (Section 5.1.3)	Inaccurate confidence interval	Few studies present
8. μ_new_ normal (Section 5.4)	Inaccurate prediction interval	Outlying studies are present

The first three assumptions in Table 3 can be avoided using one‐stage analyses and we return to this issue below. These are the hidden within‐study assumptions described in Section 3. The fourth assumption is the between‐study normality assumption described in Section 4. Assumptions 5, 6 and 7 are the three hidden assumptions made for the pooled estimate $\hat{\mu }$ described in Section 5.1. Assumption 8 is the assumption made for the true effect in a new study that is made when computing a prediction interval described in Section 5.4. Assumptions 1 and 2 are important because they have direct implications for the assumptions made about the pooled estimate. Assumptions 3 and 4 are important because together they imply that the $Y_{i}$ are normally distributed and so, for example are made in likelihood‐based analyses. The main consequence of assumptions 1–4 is that they have direct implications for the accuracy of assumptions 5, 6 and 7 that are required when making inferences about μ. The plausibility of the assumptions in Table 3 therefore cannot be considered in isolation of each other. The extent to which assumptions 4 and 8 are a source of concern is likely to depend on the context of the studies. This is because in Section 4, we found that alternatives to assuming between‐study normality have usually been motivated by the presence of outliers; in Table 3, we have indicated that this assumption is especially dubious when they are present. Hence, subject‐specific knowledge relating to the potential for unusual study results in the application area may inform the extent of concerns relating to these two assumptions.

Table 3 is intended to serve as a pertinent reminder of the key assumptions required in standard meta‐analyses and we encourage applied analysts to consider how appropriate these assumptions are in their applications. The risk of bias tool (Higgins & Green, 2011) has become a popular approach for assessing the potential for bias due to the nature of the included studies in systematic reviews. Table 3 could be used to form the basis of an analogous ‘risk of compromised statistical inference tool’. As in the usual risk of bias tool, green, yellow and red symbols could be used for each assumption to identify whether or not it is a serious source of concern. For example, in random‐effects meta‐analyses with two or three very large studies, the first three assumptions (that relate to within‐study approximations) are unlikely to be a serious source of concern, but assumption 7 will be.

### 6.1 A postulated hierarchy of sensitivity to normality assumptions

We have examined how several different forms of statistical inference may be sensitive to normality assumptions. In this section, we postulate a hierarchy of sensitivity to these assumptions in order to indicate which of these inferences can be anticipated to be the most, and least, sensitive. Our hope is that this will enable applied analysts to focus on the most serious concerns.

In terms of making inferences for μ, which are of primary interest, we propose the following hierarchy of statistical inferences that goes from the least, to the most, sensitive to departures from normality assumptions. As explained above, for both the common‐effect and random‐effects models, the point estimate $\hat{\mu }$ is simply a weighted average of the $Y_{i}$. We suggest that this is likely to be the least sensitive type of inference to departures from normality assumptions. The standard error of $\hat{\mu }$ can, in many instances, be entirely motivated without making distributional assumptions and so we suggest that this is also likely to be insensitive in this way.

Conventional confidence intervals for μ rely on the assumption that $\hat{\mu }$ is normally distributed. We can therefore anticipate that confidence intervals for μ will be more sensitive to departures from normality assumptions than the corresponding point estimates and standard errors. Prediction intervals will clearly be sensitive to the assumed distribution for the random‐effects when notable heterogeneity is present. This is because different probability distributions, despite having the same variance, can provide substantially different critical values. Hence, we anticipate that prediction intervals will in general be the most sensitive form of inference to departures from normality assumptions.

## 7 ILLUSTRATION AND DISCUSSION OF ALTERNATIVE METHODOLOGIES

Our first example nicely illustrates a situation where normal within‐study approximations, and in particular the first two hidden assumptions (Sections 3.1 and 3.2) made in conventional meta‐analysis methodologies, are best avoided. This is because the studies are small and the outcome is binary so that conventional within‐study approximations for the empirical log‐odds ratios cannot be expected to be very accurate.

As explained in Section 3.4, GLMMs are the primary proposal for avoiding the use of within‐study approximations when fitting random‐effects models. For comparative binary data this class of models includes (2) and a variety of other possibilities (Böhning et al., 2015; Jackson et al., 2018; Stijnen et al., 2010; Turner et al., 2000). However, as also explained in Section 3.4, determining which particular GLMM is most suitable in practice remains an open question. Fortunately for illustrative purposes, matters are less complicated for our first example, in the sense that all three fitted conventional random‐effects models analyses in Section 2.1 collapse to a common‐effect meta‐analysis. Hence, an obvious way to avoid the use of within‐study approximations when analysing our first example is to fit a common‐effect version of model (2) where $\tau^{2}=0$, or equivalently where $\mu_{i}=\mu$ for all *i*. This standard logistic regression provides $\hat{\mu }=0.71$ with a standard error of 0.20. The corresponding 95% confidence interval is (0.32, 1.10) and the results for this example are summarised in Table 1. Transforming the estimate and confidence interval to the odds ratio scale gives a pooled odds ratio of 2.04 (with a 95% confidence interval from 1.38 to 3.01).

Comparing these results to those in Section 2.1 ($\hat{\mu }=0.65$ with standard error 0.20), this analysis is in reasonable agreement with the conventional analysis presented above. Given the small study sizes, and so the crudeness of the conventional methods, it is perhaps surprising that the inferences from the logistic regression are so similar. However, a slightly larger estimate of treatment effect is obtained from the logistic regression, indicating that the within‐study approximations used in conventional methods have diluted the estimated treatment effect. The alternative common‐effect Mantel‐Haenszel method used in the Cochrane Review has also slightly, but to a lesser extent, diluted the estimated treatment effect.

One challenge when using more advanced methodologies, such as GLMMs, for comparative binary data is that inferences on the risk difference scale might be desired where models or computational algorithms have not implemented the identity link. In such instances, we suggest fitting a model using a logistic link and then choosing a representative baseline probability for the control group (such as the sample mean). Inferences for the risk difference, using this baseline control group probability, can then be made using the output from the fitted logistic model. Some statistical expertise is needed when adopting such an approach.

Our second example illustrates a situation where the within‐study approximations made by the standard analyses in Section 2.2 are of much less concern. Here, the studies are large and the (transformed) individual outcome data appear to be approximately normally distributed. Hence, within‐study normal approximations are acceptable. There are 40 studies so that $\hat{\mu }$ can perfectly reasonably be taken to be approximately normally distributed. Furthermore τ^2^ is well identified, so that taking the standard error of $\hat{\mu }$ as known is a reasonable approximation. One‐stage analyses of the IPD described in Section 2.2 may be considered desirable for a variety of reasons (Riley, Lambert, & Abo‐Zaid, 2010; their box 1), but the within‐study assumptions made by the conventional meta‐analyses of our second example are reasonable.

However, as noted in Section 4, there is the concern that highly heterogeneous meta‐analysis datasets like our second example may be sensitive to the distributional assumptions made for the random‐effects. This was assessed using the R package *metaplus*. This package can fit the conventional random‐effects model and also two alternative random‐effects models. In the first of these alternative models, it is assumed that the random‐effects follow a *t* distribution (Baker & Jackson, 2008), and in the second a mixture of normals is instead assumed (Beath, 2014). The *metaplus* package fits these three models using maximum likelihood and computes confidence intervals for μ using the profile likelihood (Hardy & Thompson, 1996). For all three random‐effects models (the conventional one and both alternative models), the inferences were however very similar to those in Section 2.2 (Table 2). Our second example does not appear to be sensitive to the assumed distributional form of the random effects. This further strengthens the case for the acceptability of our analyses in Section 2.2. See Böhning, Dietz, and Schlattmann (1998) for a discussion of further models and methods where mixture distributions are used, both in the context of meta‐analysis and in other application areas.

In application, it is common that aggregate‐level continuous data, rather than IPD, are available in situations where interest lies in means or mean differences. Here, the observation that the outcome data at the individual level are highly skew in some studies may discourage analysts from including these data when using standard methodologies. This is because the within‐study approximations used in these methods might then be thought to be inappropriate. However, in many instances this concern may not be warranted, because conventional meta‐analysis models assume that the $Y_{i}$, not the individual‐specific responses, is normally distributed. The CLT can often be used to motivate normal approximations for the $Y_{i}$ in situations where the individual‐level data are skew. It should however be noted that larger samples are generally needed to invoke the CLT when data are skew, so we would not wish to encourage a blasé attitude to this issue. Extending this theme a little further, in our study‐specific linear regressions shown in (1), we log‐transformed the outcome data so that these models better describe our data. However least squares estimates, and their standard errors, are valid without making normality assumptions. If interest lies in mean differences between CRP levels, then linear models as shown in (1), but using untransformed CRP_*j*_ as outcome data, could therefore be fitted and the resulting estimates and within‐study variances used in the second stage of conventional meta‐analyses. However, there are two important caveats. First, substantial efficiency could be gained by using a skew error distribution in the study‐specific linear regressions for the untransformed CRP levels. Secondly, we would not advocate one‐stage analyses that involve fitting GLMMs that assume normality to highly skew data. This is because fitting a such a mis‐specified model has the potential to result in unreliable estimates of the random‐effects distribution and hence misleading inference. Further research is needed to determine the problems associated with fitting mis‐specified random‐effects models in one‐stage IPD meta‐analyses. An alternative strategy is to proceed in a similar manner as for binary outcome data described above, where the analysis is performed using log‐transformed data and inferences on the untransformed scale are made by using a representative baseline value.

## 8 DISCUSSION

We began by asking ‘When should meta‐analysis avoid making hidden normality assumptions?’ To fully understand the issues relating to this question most of our energies have however gone into describing how this distribution is extensively used. A vague answer to our question, that we suspect that most readers will be able to agree with, is ‘Meta‐analysis should avoid using the normal distribution more often than it currently does’. In particular, it would seem reasonable to conclude that the conventional within‐study assumptions are often especially crude and should be more often explicitly avoided. However, more research is needed to understand the situations where the current conventional approach is inadequate and the implications of using alternative methodologies. Our work leaves many important issues unresolved and there are important questions that we do not claim to have the answers to. For example, in some meta‐analyses many or even most studies may be large enough for the three hidden assumptions in Section 3 to be regarded as of little concern. However, it will also often be the case that some studies are much smaller than this, and hence there is the obvious question of ‘Does it matter if just a few of the studies are too small to imply accurate within‐study normal approximations?’ This may be of particular concern in random‐effects meta‐analyses, because small studies will in general contribute more relative weight in these analyses than in common‐effect analyses. Until issues such as these are better understood, one practical use of the methodologies described in Section 7 is that they could be used as sensitivity analyses that assess how robust standard analyses are to the normality assumptions that they make.

We have two main suggestions for how current practice might improve. First, one‐stage analyses, that assume GLMMs, avoid making often crude approximations within studies and are a feasible alternative that should be considered more often. Methods based on the profile likelihood (Böhning, Kuhnert, & Rattanasiri, 2008) should also be considered more often in application. In principle, more sophisticated and alternative methods such as these are preferable, especially in situations where some, many or all studies are small, or the event of interest is rare (Böhning et al., 2015). However, GLMMs do not avoid all uses of the normal distribution and they also present alternative issues and difficulties (Jackson et al., 2018). Second, we suggest that a standard framework for communicating concerns about the statistical methods used in meta‐analyses would be a useful next step. The expectation that systematic reviewers will attempt some form of study quality or risk of bias assessment is now widely accepted, but meta‐analyses are usually presented with scant explanation of the extent to which the approximations and assumptions made by them are accurate and reasonable. We suspect that if consumers of systematic reviews were better informed about the accuracy of the approximations used in statistical methods for meta‐analysis, then the demand for more sophisticated statistical methods would increase. Table 3 could provide a basis for communicating these issues, but we accept that this just provides a starting point for discussion rather than a concrete recommendation for a ‘risk of compromised statistical inference tool’ (RoCSI tool). Such a tool may provide a useful framework for statisticians and consumers to better understand the assumptions made by meta‐analyses and so facilitate a stronger defence of these assumptions in situations where conventional meta‐analyses are appropriate.

There are many practical issues when performing meta‐analyses, including the determination of appropriate inclusion criterion and extracting suitable outcome data. These issues will in many instances be far more important than the subtle statistical nuances of the modelling that provide our focus. In applications it may well often be that concerns about inaccuracy of normal approximations are simply ‘the least of our problems’. Despite this, a better understanding of the assumptions made in meta‐analyses, when these assumptions are acceptable and what might be done when they are not, would be beneficial for many involved in performing systematic reviews.

We have focused on the simplest methods for meta‐analysis. Matters are even more complicated in multivariate meta‐analyses (Jackson, Riley, & White, 2011; van Houwelingen et al., 2002) and network meta‐analyses (Salanti, 2012) because then outcome data in the second stage of meta‐analyses can be correlated. Multivariate normal distributions are then used in the conventional methodologies for multivariate and network meta‐analysis, making additional assumptions. More sophisticated methods for modelling random effects (e.g. Kuss, Hoyer, & Solms, 2014; Nikoloulopoulos, 2015) are possible in the multivariate setting, so that the issues discussed in Section 4 become more pressing. Despite this, simplified models where all between‐study correlations are taken to be a half are typically used in network meta‐analyses, but there is also a case for considering more general models for the variance structure (Lu & Ades, 2009; White, Barrett, Jackson, & Higgins, 2012) in situations where they can be adequately identified.

To summarise, we have seen that the normal distribution is extensively used in conventional meta‐analysis methodologies. We suspect that the relatively simple and direct nature of the calculations used in meta‐analysis conceals this, but we hope that our discussion is illuminating. It is perhaps easy to criticise standard methods for meta‐analysis on the grounds that implausible normality assumptions are sometimes required, but any alternatives that avoid them should be carefully assessed before we consider them for routine use. Our suspicion is that more advanced methodologies such as GLMMs will become more common in the future, but also that these methods will ‘live alongside’ the current approach. If so, this would be in much the same way as χ^2^ tests, Fisher's exact test and tests from fitting log‐linear models co‐exist in the context of testing for an association in a single 2 × 2 table. These different statistical methods, that have the same purpose, possess different types of advantages. Important criteria for assessing statistical methods include optimality, transparency, ease of computation and intuitive appeal. For similar reasons as the χ^2^ test has endured in applied work, we suggest that the conventional methods for meta‐analysis will be continue to be adopted whilst also giving ground to alternatives that have better statistical properties in some, or possibly many, situations.

## CONFLICT OF INTEREST

The authors have declared no conflict of interest.

## Supporting information

Supplementary Material
